# Utilization of clinical practice guideline on antimicrobial in China: an exploratory survey on multilevel determinants

**DOI:** 10.1186/s12913-020-05171-z

**Published:** 2020-04-06

**Authors:** Qingwen Deng, Wenbin Liu

**Affiliations:** Department of Health Management, School of Public Health, Fujian Medical University, Room 108 in the Building for School of Public Health, 1 Xuefubei Road, Minhou District, Fuzhou, 350122 China

**Keywords:** Clinical practice guideline, Antimicrobial, Multilevel analysis, China

## Abstract

**Background:**

Nowadays, irrational use of antimicrobials has threatened public health. It’s necessary to expand the use of clinical practice guideline (CPG) on antimicrobial for facilitating the proper use of antimicrobial. However, the utilization status of CPG on antimicrobial and the influencing factors, especially the ones at the organizational level, remain largely unknown.

**Methods:**

A cross-sectional questionnaire survey was conducted on a sample among physicians from 16 public hospitals in the eastern, central and western parts of China. A multilevel regression model was employed to examine factors associated with physicians’ utilization of CPG on antimicrobial.

**Results:**

A total of 815 physicians were included in this study. About 80% of the surveyed physicians reported their adherence to the CPG on antimicrobial. Dimensions of “subjective norm”, “perceived risk” and “behavioral intention” from the domain of physician belief, a dimension of “ease of use” from the domain of CPG traits, and dimensions of “top management support” and “organization & implementation” from the domain of hospital practice were significantly associated with physicians’ utilization of CPG on antimicrobial. And except for working department, most demographics characteristics of the physician were not found to be significantly related to the CPG use. In addition, it also showed that region is a significant factor affecting physicians’ CPG use.

**Conclusions:**

This study depicted the current status of CPG on antimicrobial and comprehensively identified its potential determinants not only from the three domains at the individual level, such as physician belief, but also from the location region at the organizational level. The results will provide a direct reference for the implementation of CPG on antimicrobial.

## Background

Antimicrobial has left its marks on the history of medical treatment, owing to effectively lowering the morbidity and mortality from infectious diseases [1]. Nowadays, however, the rise in the prevalence of irrational use of antimicrobial has weakened its original effectiveness and contributed to growing drug resistance that will undermine the ability to fight infections with varying degrees [2], or even worse.

As one of the world’s largest consumers of antimicrobial [3], China has witnessed some of the most severe crises brought on by the antimicrobial resistance (AMR). To address AMR, various measures have been taken by health authorities in China [4]. Among these measures, Clinical practice guideline (CPG) is a professional recommendation for physicians basing on current best evidence, and it has been commonly reported for its significant role in standardizing clinical treatment and improving the outcomes of medical services [5]. National Health Commission of the People’s Republic of China also have issued Guiding Principles for Clinical Application of Antimicrobial Drugs in 2015, which introduced indications and precautions of various antimicrobial agents, and stated that antimicrobial should be managed in three categories, namely “unrestricted use”, “restricted use” and “special use” [6]. However, this guideline did not serve as a mandatory requirement for physicians and health institutions, and there was no regulation or policy on the compulsory implementation of CPG.

Many studies have been carried out on the status of the antimicrobial application [7–10], put forward the concerns of irrational use and drug resistance of antimicrobial. Some scholars investigated the reasons for irrational use of antimicrobial, such as the compensating scheme of health insurance, lack of knowledge in the rational use of antimicrobial, a tense doctor-patient relationship [9], etc. Meanwhile, there are also some studies focusing on the physicians’ antimicrobial prescribing behavior [7, 11–13] or the public’s self-medication with antimicrobial drugs [14–16]. And most of the studies usually assess their knowledge, attitude, and practice (KAP) on antimicrobial at the individual level [11–13, 17, 18]. However, little attention has been paid to the current status of the implementation of CPG on antimicrobial, and few studies have involved the potential influencing factors from the domains other than the individual, such as organizational impact, CPG traits and so on. To bridge this knowledge gap, this study will comprehensively include sets of potential influencing factors from the domains of physician, hospital, and CPG itself, and seek to investigate determinants associated with the utilization of CPG on antimicrobial at different domains. These results of this study can be provided as a theoretical basis to tailor interventions about promoting the utilization of CPG on antimicrobial.

## Methods

### Study design

As China is a vast country with huge regional diversity at socioeconomic development, we conducted a cross-sectional questionnaire study using a multistage sampling method. In the first stage, provinces of Fujian, Hubei, Yunnan & Sichuan were selected on behalf of eastern, central and western regions of China, respectively. Secondly, 5~6 general hospitals (including tertiary and secondary hospitals) were selected from each of the selected regions. Lastly, in each selected hospital, 16~20 physicians of tertiary hospitals and 10~15 physicians of secondary hospitals were randomly sampled from major departments of internal medicine and surgery, respectively. And 3~5 physicians of tertiary hospitals were randomly sampled from the other four sorts of departments as gynecology and obstetrics, ophthalmology and otorhinolaryngology, orthopedics, and others, respectively. While in each sampled secondary hospital, about 10 physicians were randomly selected from these four departments in total. Thus, 50~60 physicians from each tertiary hospital and 30~40 physicians from each secondary hospital were invited to participate in the survey. Ethics approval was obtained from the medical ethics committee, Fujian Medical University, China.

### Questionnaire development

The questionnaire was developed basing on the classical theoretical model of adoption behavior and technology diffusion, such as the Theory of Planned Behavior (TPB), Technology Acceptance Model (TAM), and Technology-Organization-Environment framework (TOE). TPB suggests individual behavior is ultimately influenced by behavioral intention, while the behavioral intention is affected by attitude, subjective norm and perceived behavioral control [19]. TAM refers to perceived usefulness and perceived ease of use as two crucial factors for innovation adoption [20]. TOE emphasizes not only the influence of technology itself on knowledge diffusion but also the importance of the organization environment [21], for example, support from top management and implementation practice. We integrated the three theories to guide the measurement of potential determinants of CPG utilization and also collected regarding survey items in the questionnaire applied in previous studies [19–32]. The draft of the questionnaire was further revised by experts in the fields of clinical medicine, health management, and epidemiology and health statistics.

The final structured questionnaire consisted of three parts 33 items. Part 1 covered 24 items belonging to 8 dimensions from 3 domains, namely domain of “physician belief” (including dimensions of “attitude”, “subjective norm”, “perceived risk”, “behavioral intention”), the domain of “CPG traits” (including dimensions of “relative advantage”, “ease of use”), and domain of “hospital practice” (including dimensions of “top management support”, “organization & implementation”). Part 2 covered 3 items to measure physicians’ utilization of CPG on antimicrobial. Part 3 was a personal information card with 6 items, including several basic characteristics of participants as gender, age, education, professional title, department and years of practice. Questions in part 1 and part 2 were measured using a five-point Likert scale, where 1 = Strongly disagree, 2 = Disagree, 3 = Neutral, 4 = Agree, and 5 = Strongly agree. The questionnaire showed satisfactory reliability with Cronbach’s α of each dimension and the whole questionnaire above the recommended threshold of 0.7 [33].

### Data collection

Data collected from April 2018 and lasted for nearly 1 year. With the support of sampled hospitals, each round for filling out the questionnaire was accompanied by trained facilitators to introduce the study purpose, ensuring participants’ understanding of this study and what to do. All responses were anonymous, but participants were invited to submit their contact information voluntarily if they were interested in the study and wanted to keep informed of the results. Written informed consent was obtained from all study participants.

### Analysis

Descriptive statistics were performed to depict the characteristics of physicians and hospitals. Each dimension scores were calculated as the mean item scores per dimension, and the score of the utilization of CPG on antimicrobial was obtained by taking the average of its three items. Spearman Rank correlation was used to assess the relationships between socio-demographic characteristics and utilization of CPG on antimicrobial, Pearson correlation was used to assess the relationships between each dimension and utilization of CPG on antimicrobial. In addition, given the hierarchical nature of the data [34], multilevel linear regression model (SAS University Edition) was performed to identify the association between demographic characteristics, organizational characteristics, dimension scores from three domains (independent variables) and utilization of CPG on antimicrobial (dependent variable). The rationale for using the multilevel approach in relation to the clustering effect is that physicians (individual level) in the same hospital (organizational level) tend to be more alike in their personal belief or perceptions of CPG use, as well as their assessments on CPG traits or hospital practice in promoting CPG use and real CPG utilization. Thus, the multilevel approach would be more robust in determining whether factors at the organizational level or the individual level are statistically significant. Firstly, a null model (Model 0) with neither level 1 (individual) nor level 2 (organization) determinants would conduct to identify whether the sample data has clustering effect (i.e. hierarchical structure), by calculating the intraclass correlation coefficient (ICC). Secondly, structural variables of individual-level (attitude, subjective norm, perceived risk, behavioral intention, relative advantage, ease of use, top management support, and organization & Implementation) were added to model 1. In the third and fourth steps, demographic characteristics of individual-level and organizational-level variables were added to see whether the addition of different variables improved the model, as shown in Model 2 and Model 3, respectively. Statistical significance was set at *P* < 0.05.

## Results

### Participants

A total of 815 physicians from 16 general hospitals were included in this study. The characteristics of the participants and the hospitals were presented in Table 1. Over half of the respondents were males (*n* = 459, 56.32%). Most of the respondents were under 45 years old (*n* = 728, 89.33%). Almost all of them had a bachelor’s degree or above (*n* = 802, 98.40%). With regard to the working department, 37.18% (*n* = 303) were from internal medicine and 34.97% (*n* = 285) were from the surgery department. Regarding hospital characteristics, the samples consisted of 12 tertiary hospitals and 4 secondary hospitals. And 6 hospitals were in the eastern, 5 in the central and 5 in the western of China.

**Table 1:** Characteristics of the sample physicians and hospitals

Characteristic	Frequency	Percentage (%)
Physician characteristics (*n* = 815)		
Gender		
Male	459	56.32
Female	356	43.68
Age		
< 35 years old	432	53.01
35~44 years old	296	36.32
≥ 45 years old	87	10.67
Education		
Junior college or below	13	1.60
Bachelor	345	42.33
Master	379	46.50
Doctor	78	9.57
Professional Title		
Junior	304	37.30
Intermediate	310	38.04
Senior	201	24.66
Department		
Internal medicine	303	37.18
Surgery	285	34.97
Gynecology and obstetrics	73	8.96
Ophthalmology and otorhinolaryngology	73	8.96
Other	81	9.94
Years in Practice		
< 5 years	254	31.17
5~10 years	241	29.57
11~15 years	227	27.85
16~20 years	83	10.18
> 20 years	10	1.23
Hospital characteristics (*n* = 16)		
Ranking		
Tertiary	12	75.00
Secondary	4	25.00
Region		
Eastern	6	37.50
Central	5	31.25
Western	5	31.25

### Current status of the utilization of CPG on antimicrobial

The utilization of CPG on antimicrobial was assessed by three items, which had average scores of 3.95 ± 0.62, with 1 being the minimum scores and 5 being the maximum scores. Of the statements “In the past year, I have strictly followed the CPG on antimicrobial in practice”, 81.23% (*n* = 662) of physicians strongly agreed or agreed on it, 16.69% (*n* = 136) were in neutral, and 2.09% (*n* = 17) against or strongly against it. Eighty percent of physicians (*n* = 652) reported their agreement or strong agreement on the item “In the past year, I have actively participated in the study or training of CPG on antimicrobial”, 18.04% (*n* = 147) declared their neutrality, and 1.96% (*n* = 16) have an objection or strongly objection to it. Regarding the statement of “In the past year, I have actively recommended the CPG on antimicrobial to colleagues”, 71.90% (*n* = 586) of physicians reported they have recommended the CPG on antimicrobial to other medical staff, 25.89% (*n* = 211) showed their neutrality, and 2.21% (*n* = 18) were opposed or strongly opposed to this statement.

### Perceptions and beliefs towards the utilization of CPG on antimicrobial

Physicians’ perceptions and beliefs towards the utilization of CPG on antimicrobial consisted of four dimensions, namely attitude, subjective norm, perceived risk, and behavioral intention. The mean score for each dimension was 4.29 ± 0.56, 4.15 ± 0.61, 2.24 ± 0.85, 4.13 ± 0.56, respectively (see Table 2). Most physicians reported that following the CPG on antimicrobial in clinical practice was the right thing (91.66%, *n* = 747), a wise choice (90.43%, *n* = 737) and good for all (93.99%, *n* = 766). Similarly, most physicians reported that people who were important to them (e.g. colleagues and superiors) were tended to follow CPG on antimicrobial (86.38%, *n* = 704), had a positive evaluation of CPG on antimicrobial (88.10%, *n* = 718), thought it was a right thing to use CPG on antimicrobial (89.82%, *n* = 732). The proportion of physicians who were afraid the implementation of CPG on antimicrobial would take their extra time, reduce revenue and lower efficiency, was 19.75% (*n* = 161), 9.20% (*n* = 75), 12.15% (*n* = 99), respectively. And regarding behavioral intention, the majority of physicians said they were willing to use CPG on antimicrobial (92.39%, *n* = 753), recommend it to other doctors (86.63%, *n* = 706), follow CPG on antimicrobial in the future (87.24%, *n* = 711).

**Table 2:** Scores for each dimension

Domain	Dimension	Mean	S.D.
Physician belief	Attitude	4.29	0.56
	Subjective norm	4.15	0.61
	Perceived risk	2.24	0.85
	Behavioral intention	4.13	0.56
CPG traits	Relative advantage	3.95	0.68
	Ease of use	3.86	0.65
Hospital practice	Top management support	3.99	0.63
	Organization & Implementation	4.01	0.64

### Traits of the CPG on antimicrobial

The traits of the CPG on antimicrobial were measured by two dimensions as relative advantage and ease of use. The mean value for each dimension was 3.95 ± 0.68 and 3.86 ± 0.65, respectively. With respect to the dimension of relative advantage, the items “reducing medical costs”, “improving prescribing efficiency” and “contributing to better clinical outcomes” were appreciated by 71.66% (*n* = 584), 76.93% (*n* = 627) and 78.65% (*n* = 641) of physicians, respectively. And there were also about a fifth of physicians reported their neutral stance on these items. Concerning the dimension of ease of use, 75.21% (*n* = 613) of the respondents deemed their incapability in mastering the knowledge of CPG on antimicrobial in a short time. After grasping the CPG on antimicrobial, 80.61% (*n* = 657) of the physicians perceived that they can quickly put it into practice, while 69.33% (*n* = 565) of them had a view that the CPG was simple and easy to use.

### Hospital practice in promoting the utilization of CPG on antimicrobial

The dimensions of “top management support” and “organization & implementation” were applied to depict the hospital practice in promoting the utilization of CPG on antimicrobial. The average score for each dimension was 3.99 ± 0.63 and 4.01 ± 0.64, respectively (see Table 2). Concerning the dimension of “top management support”, 83.07% (*n* = 677) and 84.05% (*n* = 685) of the physicians agreed or strongly agreed on the statements of “Administrators promoted the widely use of CPG on antimicrobial in various departments” and “Administrators attached great importance to the promotion of CPG on antimicrobial”, respectively. And three-quarters of physicians thought the administrators of the hospital had provided supports in training, funding and other aspects. A similar situation also existed in the dimension of “organization & implementation”. The proportion of the physicians reported that their working hospitals have provided information about CPG on antimicrobial, held regular feedback on the CPG use, and performed a daily inspection, supervision, and evaluation, was 81.10% (*n* = 661), 80.49% (*n* = 656) and 85.03% (*n* = 693), respectively.

### Correlations to the utilization of CPG on antimicrobial

The result of correlation analysis (Table 3) showed physicians’ utilization of CPG on antimicrobial was significantly correlated (*P* < 0.001) with the dimensions of attitude, subjective norm, perceived risk, behavioral intention, relative advantage, ease of use, top management support and organization & implementation, respectively. In addition, it also showed that age, professional title, years in practice and region were significantly associated with physicians’ utilization of CPG on antimicrobial.

**Table 3:** Factors correlated to utilization of CPG on antimicrobial

Characteristic	Correlation coefficient	*P* value
**Individual level**		
*Domain: Physician belief*		
Attitude	0.472	**< 0.001**
Subjective Norm	0.535	**< 0.001**
Perceived Risk	−0.322	**< 0.001**
Behavioral Intention	0.644	**< 0.001**
*Domain: CPG traits*		
Relative Advantage	0.568	**< 0.001**
Ease of use	0.543	**< 0.001**
*Domain: Hospital practice*		
Top management support	0.601	**< 0.001**
Organization & Implementation	0.568	**< 0.001**
*Demographics characteristics*		
Gender	0.024	0.497
Age	0.084	**0.016**
Education	−0.034	0.332
Professional Title	0.051	0.143
Department	−0.013	0.709
Years in Practice	0.081	**0.021**
**Organizational level**		
Hospital Rank	−0.020	0.565
Region	0.174	**< 0.001**

### Multilevel linear regression analysis

Table 4 presented the two-level linear regression results of the utilization of CPG on antimicrobial. The ICC coefficient of the null model was 0.062 (*P* < 0.05), suggesting the utilization of CPG on antimicrobial by individuals was clustered among the same organizations, and the two-level hierarchical structure was suitable for multilevel model analysis [35]. At the individual level, the utilization of CPG on antimicrobial was significantly associated with subjective norm (*P* < 0.001), perceived risk (*P* = 0.016), behavioral intention (*P* < 0.001) at the domain of physician beliefs, ease of use (*P* < 0.001) at the domain of CPG traits, and top management support (*P* < 0.001), organization & implementation (*P* < 0.001) at the domain of hospital practice (Model 1). Additionally, with regard to the demographic characteristics, physicians who worked at the department of ophthalmology and otorhinolaryngology had a significant reduction in the utilization behavior of CPG on antimicrobial than the ones working in other departments (Model 2).

**Table 4:** Multilevel linear regression of the utilization of CPG on antimicrobial

	Model 0	Model 1	Model 2	Model 3
Intercept	3.957***	1.796	2.306*	2.913**
**Individual level**				
*Domain: Physician belief*				
Attitude		− 0.017 (− 0.087~0.053)	−0.010 (− 0.080~0.061)	−0.019 (− 0.090~0.052)
Subjective norm		0.155*** (0.089~0.221)	0.151*** (0.087~0.214)	0.158*** (0.095~0.221)
Perceived risk		−0.048** (− 0.085~ − 0.012)	−0.038* (− 0.074~ − 0.001)	− 0.045** (− 0.082~ − 0.009)
Behavioral intention		0.304*** (0.226~0.382)	0.297*** (0.221~0.373)	0.297*** (0.221~0.372)
*Domain: CPG traits*				
Relative advantage		0.05 (− 0.022~0.121)	0.046 (−0.169~0.109)	0.038 (− 0.025~0.101)
Ease of use		0.127*** (0.067~0.188)	0.124*** (0.066~0.182)	0.124*** (0.067~0.182)
*Domain: Hospital practice*				
Top management support		0.167*** (0.100~0.234)	0.165*** (0.098~0.233)	0.159*** (0.091~0.226)
Organization & Implementation		0.145*** (0.079~0.212)	0.166*** (0.103~0.229)	0.161*** (0.098~0.223)
*Demographic characteristics*				
Gender (Ref: Female)				
Male			0.011 (−0.053~0.074)	0.030 (−0.034~0.094)
Age (Ref: ≥45 years old)				
< 35 years old			−0.002 (− 0.198~0.193)	0.025 (− 0.169~0.220)
35~44 years old			0.065 (−0.107~0.238)	0.087 (− 0.085~0.259)
Education (Ref: Doctor)				
Junior college or below			0.114 (−0.142~0.371)	0.069 (−0.193~0.332)
Bachelor			0.041 (−0.069~0.151)	0.016 (−0.098~0.130)
Master			0.027 (−0.077~0.130)	0.018 (−0.084~0.120)
Professional Title (Ref: Senior)				
Junior			−0.464 (− 0.183~0.090)	−0.051 (− 0.186~0.085)
Intermediate			−0.006 (− 0.104~0.091)	−0.017 (− 0.113~0.080)
Department (Ref: Other)				
Internal medicine			−0.035 (− 0.139~0.069)	−0.013 (− 0.115~0.090)
Surgery			−0.039 (− 0.145~0.066)	−0.019 (− 0.124~0.086)
Gynecology and obstetrics			0.002 (−0.131~0.136)	0.022 (−0.111~0.155)
Ophthalmology and otorhinolaryngology			−0.171* (− 0.303~ − 0.038)	−0.160* (− 0.292~ − 0.029)
Years in Practice (Ref: > 20 years)				
< 5 years			− 0.263 (− 0.601~0.075)	−0.284 (− 0.620~0.052)
5~10 years			−0.275 (− 0.602~0.051)	−0.289 (− 0.614~0.035)
11~15 years			−0.294 (− 0.608~0.019)	−0.303 (− 0.615~0.008)
16~20 years			−0.181 (− 0.456~0.095)	−0.173 (− 0.447~0.101)
**Organizational level**				
Hospital rank (Ref: Secondary)				
Tertiary				0.003 (−0.084~0.089)
Region (Ref: Western)				
Eastern				−0.141*** (− 0.223~ − 0.060)
Central				−0.068 (− 0.149~0.013)
Random effects^a^				
Organization, variance	0.364 (0.018)***	0.162 (0.008)***	0.166 (0.008)***	0.164 (0.008)***
-2 Log likelihood	1515.258	930.912	940.728	941.152

Notes: Presented by *t* value or odds ratio and its 95% confidence intervals; * *P* < 0.05, ** *P* < 0.01, *** *P* < 0.001;

^a^ Estimate and SE

As for the organizational level, the multilevel regression analysis showed that the utilization of CPG on antimicrobial was less among the physicians in the eastern hospitals, while compared with their peers in the western hospitals (*P* = 0.001).

## Discussion

Since there was a dearth of literature on the utilization of CPG on antimicrobial and its potential determinants, this study described the current status of CPG utilization and comprehensively identified its potential influencing factors from the domains of physician belief, CPG traits, and hospital practice. The results of this study will not only provide direct guidance for the implementation of CPG on antimicrobial, but also add the research knowledge and evidence on CPG use, and further provide a beneficial reference for expanding CPG impact.

As demonstrated in this study, about 80 % of the surveyed physicians reported their strict adherence to the CPG on antimicrobial and active participant in regarding training, and more than 70 % of the participants have recommended the CPG on antimicrobial to their colleagues. These results indicated a fairly high utilization level of CPG on antimicrobial. Many physicians had implemented the CPG in their work and actively took their efforts to expand its use.

The ICC coefficient indicated the between-hospital differences (i.e. organizational-level factors) contributed to explaining the variance of 6.2% in physicians’ utilization of CPG on antimicrobial, which has exceeded the recommended threshold of 0.059 for the requirement of multilevel analysis [35]. However, it was still a low ICC, which is consistent with the findings of previous studies that high ICC was rare in standardized medical settings [36].

With respect to the determinants identification, dimensions of “subjective norm”, “perceived risk” and “behavioral intention” from the domain of physician belief, the dimension of “ease of use” from the domain of CPG traits, and dimensions of “top management support” and “organization & implementation” from the domain of hospital practice showed significant association with physicians’ CPG use. Physicians who worked in the different departments were significantly different in their utilization of CPG on antimicrobial, while other demographic characteristics of the physician were not found to be significantly related to the CPG use. Besides, for the potential influencing factors of the hospital characteristics, the association between hospital rank and the CPG use was not found, while the region was a significant factor as there was less utilization of CPG on antimicrobial among physicians in the eastern region. The plausible reason may be that the guideline utilization behavior of physicians, to some extent, were shaped by the sociocultural and socioeconomic context of the regions [37, 38]. And the autonomous behaviors were more apt to be tolerated in the regions with advanced economic and social development, such as the eastern part of China.

Consistent with many previous researches, this study also highlighted the importance of organizational implementation and management support [23, 39, 40]. With regard to promoting the use of CPG on antimicrobials, the “organization & implementation” activities (routine information collection, inspection, supervision, evaluation, feedback, etc.) will greatly benefit shaping expected behavior and norms of the medical staff. And the concrete support (information, education, funds, personnel, etc.) from hospital administrators also expects to play a positive role in leading a smooth process of CPG uptake. Besides, having been deemed as powerful predictors in former studies, many factors on physician belief also demonstrated their significant effect on the use of CPG on antimicrobial in this research. Such as subjective norm, a kind of perceived norm and pressure from influential persons, it is unsurprisingly for its significant association with CPG use detected in this study. Especially in the setting of public hospitals with a clear hierarchical system [41], it seems inevitable for the individual to subject to invisible pressure from all around like colleagues or superiors, and comply with perceived norms and orders [42], such as strict adherence to the CPG or greater emphasis on physician autonomy. This point was also mutually verified with the previous content that a significant impact of organizational activities on CPG use. Additionally to the hospital practice and physician belief, the impact of CPG traits on CPG use also can’t be ignored. This study also revealed that physicians tend to adopt the CPG easy to master and use. To avoid taking the first-line physicians too much time and effort to learn CPG and put it into practice, it will be wise to concise the CPG on antimicrobials as much as possible. And some brief explanations of the key points will be probably appreciated and further benefit expanding its use.

However, contrary to previous researches [28, 41], significant effects of attitude and relative advantage were not detected in this study. The plausible reason may be that many physicians were influenced by external pressure whatever their subjective attitude or assessment towards certain guideline [27, 41]. These findings also further confirmed the impacts of subjective norm and perceived risk. Additionally, there were also other contraries to former papers that younger physicians or with fewer years’ experience tended to be more likely to use guidelines [43, 44]. Such a difference may have resorted to the situation that physicians in East Asia be easily “institutionalized” when they first enrolled in the working hospital. These results implied the great importance of organizational impact, which also explained the phenomena reported in the current studies that physicians working in different departments demonstrated great differences in compliance with CPG [45].

Generally speaking, the results of this study will not only guide the real practice of promoting the use of CPG on antimicrobials, but also intend to provide clues or inspirations for future research. As the largest developing country with the largest population, China’s experience in promoting the use of CPG on antimicrobials will provide a helpful reference for other countries, especially most of the developing countries in the world. In additional to its significance, this study was also strengthened by some features. For example, a multilevel analysis model is applied in the data analysis to address cluster bias, which will be more robust in investigating the potential influencing factors from different levels. However, there are still some limitations to this study. First of all, owing to the social desirability bias [46], the participants of this study may be unwilling to voice negative assessments about themselves and the hospitals, which may directly lead to overestimation of the attitude, belief, intention, and utilization of CPG on antimicrobial [47]. Secondly, because of the sampling error, although the provinces included in this study was randomly selected from the eastern, central and western part of China, it was difficult to balance the number of provinces from the south and north. Similar situation also existed in the sampling hospitals that the number of selected tertiary and secondary hospitals was not balanced well. Since the clustering effects were found at the organizational (hospital) level, it was hard to eliminate this bias to the results. Thirdly, this study is also limited by collecting cross-sectional data at a single point-in-time to determine the influencing factors, it may be more prudent to investigate the causality by panel session data or so on in future research.

## Conclusions

This study contributed to the knowledge about the status of physicians’ utilization of CPG on antimicrobial and its determinants. The findings of this study revealed the significance of individual-level and organizational-level factors to promote the utilization of CPG on antimicrobial. Although the main influence was on the individual-level factors, the differences in physicians’ utilization of CPG on antimicrobial between hospitals were also found. One of the implications of this study is that the utilization of CPG on antimicrobial can be promoted by reinforcing subjective norm and ease of use, improving organizational support, and lowering perceived risk, which can provide a reference for future research about the CPG’s implementation.

## Supplementary information

**Additional file 1:.** Table 1 Reliability results of the questionnaire

**Additional file 2:.** Research questionnaire of attitudes, utilisation and its determinants of CPG on antimicrobial among physicians in China

## Data Availability

The datasets generated during and/or analyzed during the current study are available from the corresponding author on reasonable request.

## Abbreviations

CPG: Clinical practice guideline
AMR: Antimicrobial resistance
