# Supplementary material for: Utilization of clinical practice guideline on antimicrobial in China: an exploratory survey on multilevel determinants
Source: BMC Health Serv Res. 2020 Apr 6;20:282. doi: 10.1186/s12913-020-05171-z (PMC7137508; doi:10.1186/s12913-020-05171-z)
Supplement: Supplementary file 1 — Additional file 1:. Table 1 Reliability results of the questionnaire [file 12913_2020_5171_MOESM1_ESM.doc]

Table 1 Reliability results of the questionnaire

| Dimension | Item | Cronbach's alpha | Corrected Item-Total Correlation | Cronbach's alpha if Item Deleted |
| --- | --- | --- | --- | --- |
| Attitude | ATT1 | 0.860 | 0.695 | 0.842 |
|  | ATT2 |  | 0.757 | 0.783 |
|  | ATT3 |  | 0.756 | 0.786 |
| Subjective norm | SN1 | 0.890 | 0.751 | 0.876 |
|  | SN2 |  | 0.819 | 0.814 |
|  | SN3 |  | 0.789 | 0.842 |
| Perceived risk | PR1 | 0.821 | 0.625 | 0.807 |
|  | PR2 |  | 0.704 | 0.725 |
|  | PR3 |  | 0.700 | 0.728 |
| Behavioral intention | BI1 | 0.857 | 0.719 | 0.812 |
|  | BI2 |  | 0.770 | 0.763 |
|  | BI3 |  | 0.708 | 0.822 |
| Relative advantage | RA1 | 0.852 | 0.693 | 0.828 |
|  | RA2 |  | 0.745 | 0.772 |
|  | RA3 |  | 0.740 | 0.782 |
| Ease of use | EOU1 | 0.869 | 0.748 | 0.818 |
|  | EOU2 |  | 0.759 | 0.814 |
|  | EOU3 |  | 0.753 | 0.814 |
| Top management support | TMS1 | 0.846 | 0.700 | 0.799 |
|  | TMS2 |  | 0.696 | 0.809 |
|  | TMS3 |  | 0.752 | 0.748 |
| Organization & implementation | OI1 | 0.885 | 0.756 | 0.854 |
|  | OI2 |  | 0.825 | 0.797 |
|  | OI3 |  | 0.752 | 0.859 |
| Utilization behavior | UB1 | 0.862 | 0.685 | 0.854 |
|  | UB2 |  | 0.748 | 0.796 |
|  | UB3 |  | 0.785 | 0.761 |
| The whole questionnaire |  | 0.904 |  |  |
