# Supplementary material for: Utilization of clinical practice guideline on antimicrobial in China: an exploratory survey on multilevel determinants
Source: BMC Health Serv Res. 2020 Apr 6;20:282. doi: 10.1186/s12913-020-05171-z (PMC7137508; doi:10.1186/s12913-020-05171-z)
Supplement: Supplementary file 2 — Additional file 2:. Research questionnaire of attitudes, utilisation and its determinants of CPG on antimicrobial among physicians in China [file 12913_2020_5171_MOESM2_ESM.doc]

**Research questionnaire of attitudes, utilisation and its determinants of CPG**

**on antimicrobial among physicians in China**

**Part 1. Structural Scales**

Notes. There are 5 numbers (1, 2, 3, 4, 5) on the right side of each item, where “1” means “Strongly disagree”, “2” means “Disagree”, “3” means “Neutral”, “4” means “Agree”, and “5” means “Strongly agree”. Please tick or circle the number that best fits your real feelings on the item.

**Domain: Physician belief**

| ***Attitude*** | | | | | |
| --- | --- | --- | --- | --- | --- |
| I think it’s a right thing to follow the CPG on antimicrobial. | 1 | 2 | 3 | 4 | 5 |
| I think it’s a wise choice to follow the CPG on antimicrobial. | 1 | 2 | 3 | 4 | 5 |
| I think it’s good for all to follow the CPG on antimicrobial. | 1 | 2 | 3 | 4 | 5 |
| ***Subjective norm*** | | | | | |
| People who are important to me tend to follow CPG on antimicrobial. | 1 | 2 | 3 | 4 | 5 |
| People who are important to me have a positive evaluation of CPG on antimicrobial. | 1 | 2 | 3 | 4 | 5 |
| People who are important to me think it’s a right thing to use CPG on antimicrobial. | 1 | 2 | 3 | 4 | 5 |
| ***Perceived risk*** | | | | | |
| I am afraid the grasp of CPG on antimicrobial will take extra time. | 1 | 2 | 3 | 4 | 5 |
| I am afraid prescribing via CPG on antimicrobial will reduce revenue. | 1 | 2 | 3 | 4 | 5 |
| I am afraid prescribing via CPG on antimicrobial will reduce efficiency. | 1 | 2 | 3 | 4 | 5 |
| ***Behavioural intention*** |  |  |  |  |  |
| I am willing to use CPG on antimicrobial. | 1 | 2 | 3 | 4 | 5 |
| I will follow the CPG on antimicrobial in the future. | 1 | 2 | 3 | 4 | 5 |
| I am willing to recommend CPG on antimicrobial to other doctors. | 1 | 2 | 3 | 4 | 5 |

**Domain: CPG traits**

| ***Relative advantage*** | | | | | |
| --- | --- | --- | --- | --- | --- |
| Using CPG on antimicrobial can reduce medical costs. | 1 | 2 | 3 | 4 | 5 |
| Using CPG on antimicrobial can improve prescribing efficiency. | 1 | 2 | 3 | 4 | 5 |
| Using CPG on antimicrobial can better clinical outcomes. | 1 | 2 | 3 | 4 | 5 |
| ***Ease of use*** | | | | | |
| Can master the knowledge of CPG on antimicrobial in a short time. | 1 | 2 | 3 | 4 | 5 |
| Can quickly put into practice after grasping CPG on antimicrobial. | 1 | 2 | 3 | 4 | 5 |
| It's simple and easy to use CPG on antimicrobial in practice. | 1 | 2 | 3 | 4 | 5 |

**Domain: Hospital practice**

| ***Top management support*** | | | | | |
| --- | --- | --- | --- | --- | --- |
| Administrators promote the widely use of CPG on antimicrobial in various departments. | 1 | 2 | 3 | 4 | 5 |
| Administrators provide supports in training, funding, etc. | 1 | 2 | 3 | 4 | 5 |
| Administrators attach great importance to the promotion of CPG on antimicrobial. | 1 | 2 | 3 | 4 | 5 |
| ***Organization & Implementation*** | | | | | |
| The hospital provides information about CPG on antimicrobial. | 1 | 2 | 3 | 4 | 5 |
| The hospital performs daily inspection, supervision and evaluation. | 1 | 2 | 3 | 4 | 5 |
| The hospital holds regular feedback on the use of CPG on antimicrobial. | 1 | 2 | 3 | 4 | 5 |

**Utilisation behaviou**r of the CPG on antimicrobial

| In the past year, I have strictly followed the CPG on antimicrobial in practice. | 1 | 2 | 3 | 4 | 5 |
| --- | --- | --- | --- | --- | --- |
| In the past year, I have actively participated in the study or training of CPG on antimicrobial. | 1 | 2 | 3 | 4 | 5 |
| In the past year, I have actively recommended the CPG on antimicrobial to colleagues. | 1 | 2 | 3 | 4 | 5 |

**Part 2. Personal Information Card**

1. Please choose your gender.

A. Male B. Female

2. Please write down your age: ________

3. Please choose your educational Level.

A. Junior college or below B. Bachelor C. Master D. Doctor

4. Please choose your professional Title.

A. Junior B. Intermediate C. Senior

5. Please choose your working department.

A. Internal medicine B. Surgery C. Gynaecology and obstetrics

D. Ophthalmology and otorhinolaryngology E. Orthopaedics F. Other

6. Please choose your years in practice.

A. <5 years B. 5~10 years C. 11~15years D. 16~20 years E. >20 years
